# Influence of Monomer Ratios on Molecular Weight Properties and Dispersing Effectiveness in Polycarboxylate Superplasticizers

**DOI:** 10.3390/ma13041022

**Published:** 2020-02-24

**Authors:** Huiqun Li, Yan Yao, Ziming Wang, Suping Cui, Yali Wang

**Affiliations:** 1 College of Materials Science and Engineering, Beijing University of Technology, Beijing 100124, China; lihuiquncbm@sina.com (H.L.); cuisuping@bjut.edu.cn (S.C.); wangyali1978@bjut.edu.cn (Y.W.); 2 State Key Laboratory of Green Building Materials, China Building Materials Academy, Beijing 100024, China; yaoyan@cbmamail.com.cn; 3 National Engineering Labaratory for Industrial Big-data Application Technology, Beijing 100124, China

**Keywords:** polycarboxylate superplasticizer, monomer ratio, molecular weight, dispersing effectiveness

## Abstract

A series of polycarboxylate superplasticizer (PCE) polymers were synthesized from acrylic acid (AA) and isoprenyloxy polyethylene glycol ether (IPEG) at the mole ratios of 3.0, 4.2, 5.0 and 6.0. In this study, the molecular weight properties of PCE polymers were recorded by size exclusion chromatography with the time interval of 1 h. Mini slump test was used to detect the dispersing effectiveness of PCE polymer in cement paste. The results indicated that the reaction rate of monomers, conversion of 52IPEG macromonomer and molecular weight of PCE polymers increased with the general adding ratio of AA to IPEG macromonomers while the side chain density of PCE polymers decreased. PCE polymers possessed molecular weight around 30,000 g/mol with low side chain density, and long main chain length presented high initial dispersing effectiveness at the low dosage around 0.12%. The majority of effective PCE polymers were formed during the adding period of acrylic acid in the first 3 h.

## 1. Introduction

Polycarboxylate superplasticizers (PCE) provide concrete high fluidity, strength and excellent durability at low content, which efficiently promotes the development of construction industry [1,2,3]. The structure of PCEs such as functional groups, monomer sequence, molecular weight property, length of main chain and density of side chain directly affect the fluidity of cement paste. The long main chain length of PCE provides polymer with enough adsorption groups onto the cement particles while the high density of side chain provides strong hindrance steric between the surface of cement particles [4,5,6,7,8]. The optimal PCE requires the content and arrangement equilibrium of anchor and steric hindrance groups, which exhibited as monomer sequence along the backbone. PCE polymers with high feeding ratios of unsaturated carboxylic acid and macromonomer own higher ratio of AAA and AAE monomer sequences (A represented acrylic acid, E represented isoprenyloxy polyethylene glycol ether) [9] in PCE polymers. The influence of molecular structures of PCE on the fluidity and hydration process of cement paste has always been the research hotspot. [10,11,12,13]. High content of unsaturated carboxylic acid may lead the hydration delay of cement paste. On the other side, it provides high charge density and adsorption driving force for PCE polymers. Meanwhile, high side chains density should be guaranteed to supply PCE polymers with strong steric hindrance. The kinetic chain length ν presented in Formula (1) is a decisive factor of molecular weight for polymers in aqueous free radical polymerization, and it is directly proportional to the monomer concentration [*M*] but inversely proportional to the square root of initiator concentration [*I*]. The kinetic chain length ν would vary with the monomer concentration [*M*] exclusively under the fixed initiator concentration and reaction temperature. Here, *k_d_*, *k_p_* and *k_t_* are designated as chain initiation rate constant, chain propagation rate constant and chain termination rate constant, respectively.

(1) $$\nu =\frac{k_{p}}{2{\left(fk_{d}k_{t}\right)}^{1/2}}\times \frac{\left[M\right]}{{\left[I\right]}^{1/2}}$$

The monomer composition *F* in polymer is affected by the mole ratio *f* and reactivity ratio *r* of monomers in binary copolymerization according to Formula (2). The reaction of PCE is non-ideal copolymerization, for the reactivity ratio of unsaturated carboxylic acid *r*_1_ is greater than 1 while the reactivity ratio of polyethylene glycol macromonomer *r*_2_ is less than 1. Additionally, the steric hindrance effect caused by the high content of ethylene glycol in macromonomer decreases the reaction rate of polymerization, which causes the major isoprenyloxy polyethylene glycol ether (IPEG) macromonomer synthesized via comopolymerization rather than homopolymerization. Therefore, all the IPEG macromonomer is added into the reaction system at the beginning while unsaturated carboxylic acid is dropped added in a typical synthesis of PCE. Thus, the concentration ratio of IPEG macromonomer to acrylic acid decreased because of the successive consumption of macromonomer and adding of unsaturated carboxylic acid. PCE polymers with diverse molecular weight and side chain density formed as the transformation of dissociative monomer concentration and instantaneous completion of free radical copolymerization process [14,15,16,17,18].

(2) $$F_{1}=\frac{r_{1}f_{1}^{2}+f_{1}f_{2}}{r_{1}f_{1}^{2}+f_{1}f_{2}+r_{2}f_{2}^{2}}$$

It was a goal of the study to distinguish the differences of molecular properties and reaction rates of PCE polymers with different feeding ratio of monomers. A series of PCE samples were synthesized from acrylic acid and isoprenyloxy polyethylene glycol ether via aqueous free radical copolymerization at different mole ratios (*A*/*E* value). The molecular weights, mass fraction of polymers, transformation of side chain density and main chain length of PCE polymers were all derived by size exclusion chromatography (SEC) data. Apparently, the molecular structures of PCE samples have obvious correlation with the general monomer ratios added as well as their feeding speed. PCE polymers with high *A/E* value showed higher content of acrylic acid and molecular weight. The molecular weight of polymers decreased as the feeding of acrylic acid. The conversion of macromonomer increased with the *A/E* ratio. The effective PCE polymers were produced in the first 3 h. The influence of molecular properties of PCE polymers on the dispersing effectiveness in cement paste was also discussed in this study. Thus, PCE polymers with different molecular structures and dispersing effectiveness could be optimized.

## 2. Materials and Methods

### 2.1. Materials

The isoprenyloxy polyethylene glycol ether containing 52 ethylene oxide (EO) units with the molecular weight around 2400 g/mol (52IPEG) was provided by Oxiranchem, Liaoning, China. IPEG containing long chain were adopted in this study for the short type was too sensitive to the mole ratios of unsaturated carboxylic acid to IPEG macromonomers. Acrylic acid (AA), ammonium persulfate (APS) and 3-mercaptopropionic acid (3-MPA) were all purchased from Sigma-Aldrich Chemie GmbH, Milwaukee, Germany.

The cement CEM I 52.5 used for the mini slump test was supplied by Heidelberg Cement, Germany. The phase composition detected by quantitative X-ray diffraction including Rietveld refinement (Bruker axs D8) is shown in Table 1.

### 2.2. Methods

#### 2.2.1. Preparation of 52IPEG Series PCE Polymers

52IPEG series PCE polymers were synthesized by acrylic acid and 52IPEG macromonomer at the mole ratios of 3.0, 4.2, 5.0 and 6.0 via aqueous free radical copolymerization. The mole ratio of the reactants in 52IPEG3.0 sample 52IPEG: AA: APS: 3-MPA was 1.0:3.0:0.17:0.26. A typical preparation method of 52IPEG3.0 was described as following: 52IPEG solution was placed in a flask immersed in a water bath at the temperature of 60 °C. AA and 3-MPA was dissolved by deionized (DI) water and designated as solution A, while APS was dissolved by DI water and designated as solution B. Solutions A and B were dropped into the flask continuously for 3 h and 2 h preservation were still needed after the feeding of solutions. Finally, the reaction system was cooled down to 45 °C and adjusted to around pH 7.5 by base solution. 52IPEG3.0 at a solid content of 39.5% was obtained.

#### 2.2.2. Size Exclusion Chromatography

52IPEG series PCE polymers with different sizes were separated by a 2695 Separation Module with three Ultrahydrogel^TM^ columns 120, 250 and 500 from Waters, Eschborn, Germany. The molecular weight, polydispersity index and hydrodynamic radius of 52IPEG series PCE were detected by a three-angle static light scattering detector from Wyatt Technology Corp., Santa Barbara, USA. Meanwhile, the concentrations of different compositions were measured by a differential refractive index (RI 2424) detector from Waters, Eschborn, Germany. The 52IPEG series PCE was dissolved to the testing concentration of 10 g/L by DI water. A 0.1 N NaNO_3_ solution at the pH of 12.0 was used as the eluent. The *dn*/*dc* value (refractive index increment) of polyethylene glycol (0.135 mL/g) was fixed as the value of 52IPEG series PCE.

#### 2.2.3. Fluidity of Cement Paste

The fluidity of cement paste added PCE was carried out according to DIN EN 1015 at a w/c of 0.3. The dosages of PCE were recorded when the fluidity of cement paste achieved 26 ± 0.5 cm to compare the dispersing effectiveness. The vicat cone mold filling the cement paste was 40 mm height, 70 mm top diameter and 80 mm bottom diameter.

## 3. Results

### 3.1. Molecular Properties over Time

**Figure 1:**
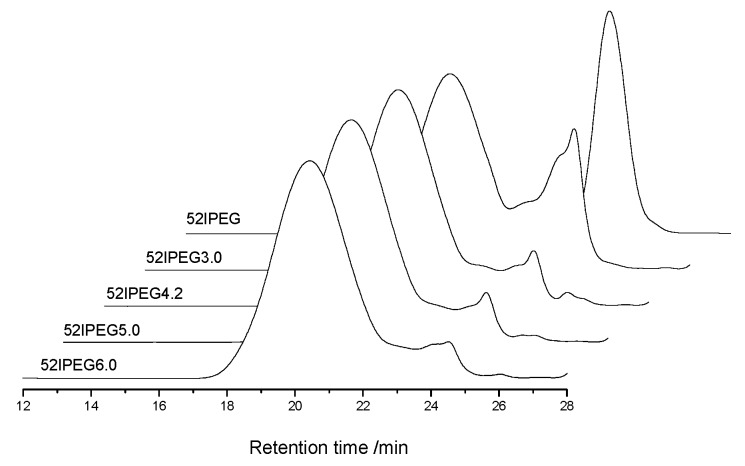
SEC (size exclusion chromatography) spectra of 52IPEG series polycarboxylate superplasticizer (PCE) at different feeding ratios of *A* to *E*.

There was no obvious split and show narrow molecular weight distribution for the final products of 52IPEG series PCE polymers in the SEC spectra with *A/E* among 3.0 and 6.0 exhibited in Figure 1. The SEC spectra of 52IPEG4.2, 52IPEG5.0 and 52IPEG6.0 PCE samples were quite similar. The mass fraction of residual macromonomer in 52IPEG3.0 was much higher than other samples. There were two reasons for the low conversion of 52IPEG macromonomer in 52IPEG3.0: firstly, the macromonomer inclines to copolymerization rather than homopolymerization, and it cannot be reacted completely at a low content of acrylic acid; secondly, the strong steric hindrance effect of EO units decreased the reaction rate on a certain extent [19]. It indicated that, the high value of *A/E* increased the final conversion of 52IPEG macromonomer.

**Figure 2:**
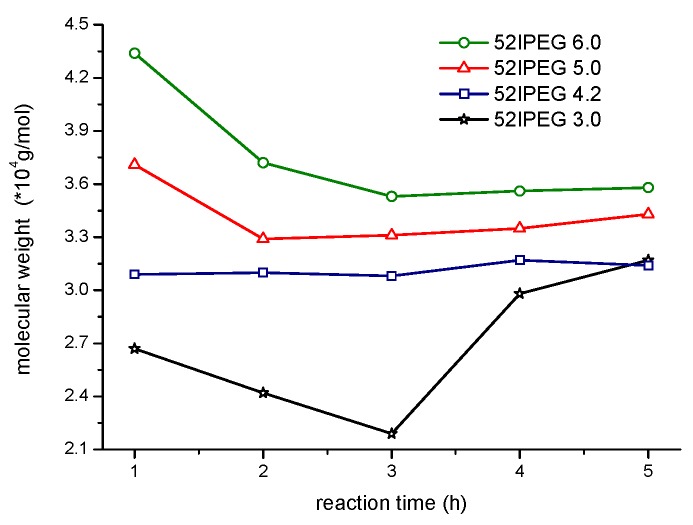
Molecular weight of 52IPEG PCEs via SEC spectra.

Generally, the molecular weight of 52IPEG series PCE polymers decreased during the adding of acrylic acid in the first 3 h and was followed by steady curves for the last 2 h as shown in Figure 2. The highest concentration of macromonomer occurred at the very beginning of the reaction for the 52IPEG series polymers. The continuous consumption of IPEG macromonomer and addition of AA caused the increase of *A*/*E* [20,21]. It could be inferred that the density of side chain in PCE polymers was decreasing in the first 3 h according to Formula (2). The molecular weight of 52IPEG was 2400 g/mol and about 33 times that of AA, so 52IPEG macromonomer contributed the major molecular weight of PCE polymer. It illustrated the molecular weight rise of 52EIPGE3.0 polymers in the last 2 h caused by the high mass ratio of macromonomer. The flat curve of 52IPEG4.2, 52IPEG5.0 and 52IPEG6.0 in the last 2 h suggested that polymers produced contained few macromonomers. In the case of 52IPEG4.2, its molecular weight did not fluctuate within 5 h, and its density of side chain decreased according to the above analysis; as a result, its main chain length was increasing during the entire reaction process.

**Figure 3:**
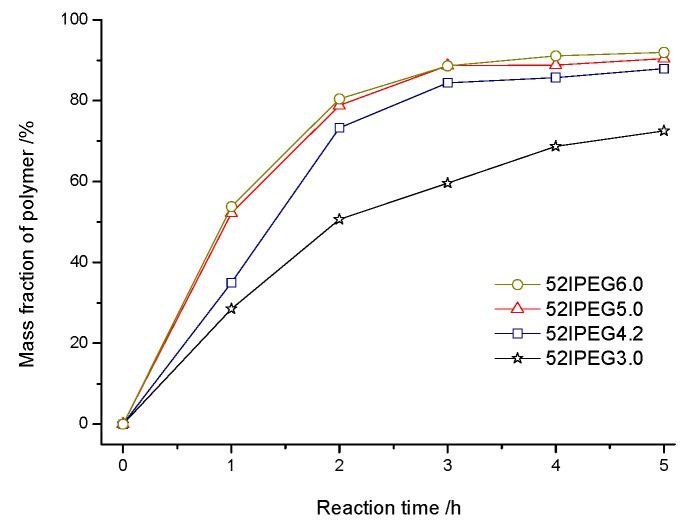
The mass fraction of polymers produced in 52IPEGs over time.

The concentration of monomers has been changing all through the reaction, which brings the continuous variation of polymer structures. The molecular weight of polymer climbed rapidly in the first 3 h before the feeding of AA finished according to Figure 2. There were rare PCE polymers produced in the following reaction except 52IPEG3.0. The general feeding ratio of acrylic acid to IPEG macromonomer improved the conversion of IPEG as well as the polymerization rate according to Figure 3. The curves of 52IPEG5.0 and 52IPEG6.0 almost coincided in Figure 1 and Figure 3, while they showed different molecular weights in Figure 2, which means similar conversion of macromonomers yet different polymer structures.

**Figure 4:**
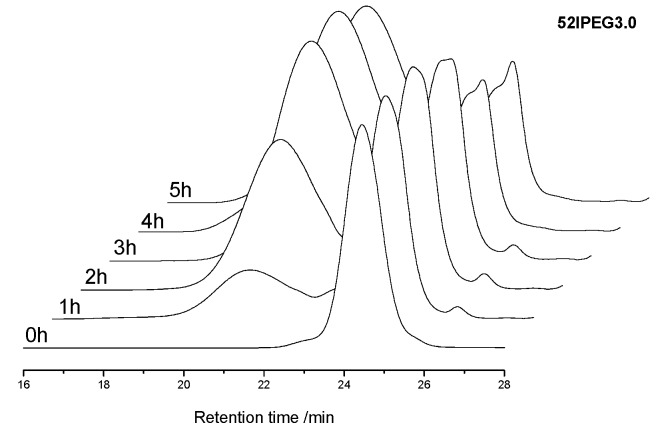
SEC spectra of 52IPEG3.0 with the time interval of 1 h.

The molecular weight properties of 52IPEGs were recorded by SEC spectra with the time interval of 1 h. Here, the samples were not separated from the polymers produced before. The polymer SEC spectra revealed the variation of reaction rate of 52IPEG3.0 in different periods in Figure 4. Polymers occupied the mass fraction of 50.6% of the system after 2 h’ reaction; then the reaction slowed down, and the total value for the last 3 h declined to around 20%. There was still a high content (27.5% mass fraction) of residual macromonomers in 52IPEG3.0 after 5 h’ reaction. The content of macromonomer was excessive and much higher than the other samples during the entire reaction process, which led the highest side chain density of the polymers. Combining with Figure 2, PCE polymers of 52IPEG3.0 possessed high side chain density, low molecular weight and short main chain length. It also proved that the reactivity ratio of acrylic acid was higher than that of 52IPEG macromonomer [22].

**Figure 5:**
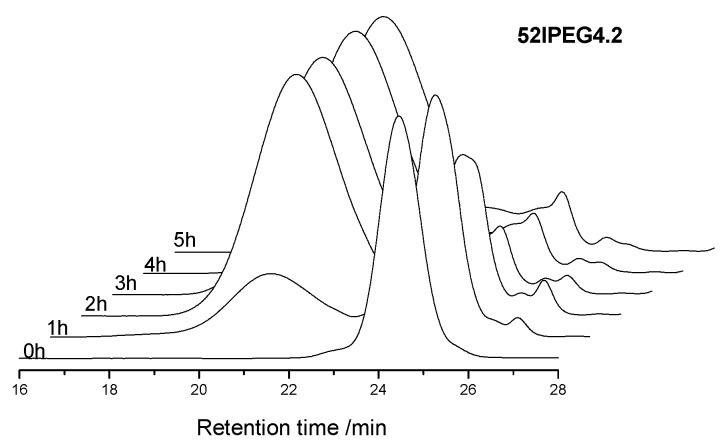
SEC spectra of 52IPEG4.2 for the time interval of 1 h.

A higher reaction rate of 52IPEG4.2 was presented in Figure 3 and Figure 5. The mass fraction of polymers already achieved 73.3% in the first 2 h, which was approximately 20% higher than that of 52IPEG3.0. It means that the high ratio of acrylic acid improved the reaction rate as well as the conversion of macromonomers in the early reaction period. Similar mass fraction of 52IPEG4.2 polymers was detected during the 3rd and 5th hour, and the polymerization was almost stagnated after the addition of AA finished. On the basis of 52IPEG4.2: (1) The main polymerization was almost completed in the first 3 h according to Figure 3, (2) the conversion of macromonomers after the 2nd hour was so far below the first 2 h, (3) the concentration of macromonomers was decreasing during the reaction, (4) the molecular weight of 52IPEG4.2 kept stable during the entire reaction process. It could be concluded that, the side chain density of 52IPEG4.2 decreased, the main chain length increased as the reaction proceeded and quite few macromonomers contained in the polymers produced after 2 h.

**Figure 6:**
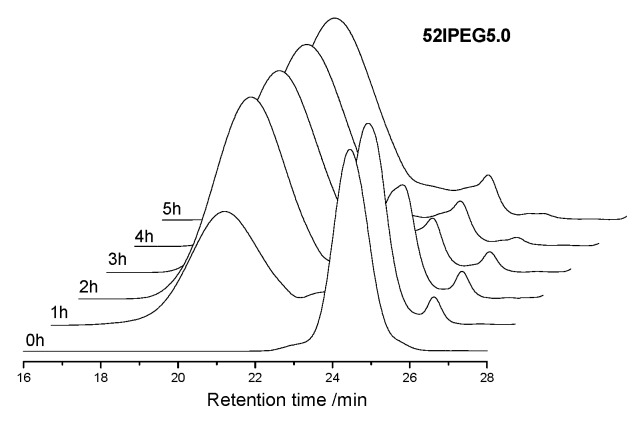
SEC spectra of 52IPEG5.0 with the time interval of 1 h.

**Figure 7:**
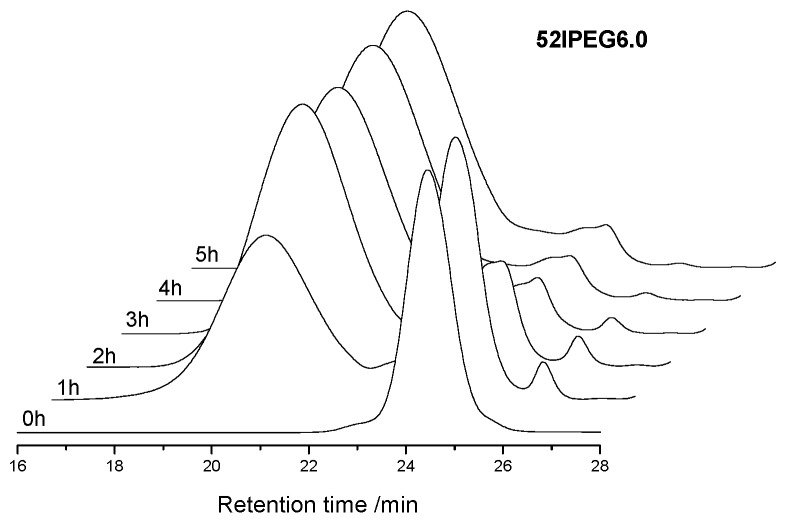
SEC spectra of 52IPEG6.0 with the time interval of 1 h.

An extremely high mass fraction (52.2%) of polymers in 52IPEG5.0 was detected for the 1st hour in Figure 6, which was around 40% and 20% higher than that of 52IPEG3.0 and 52IPEG4.2, respectively). The polymerization rate of PCE was quite sensitive to the adding amount of acrylic acid in the 1st hour. Thus, it was a critical period to control the reaction rate and polymer structures of PCE polymers [23,24,25]. The difference of the mass fraction of 52IPEG4.2 and 52IPEG5.0 polymers was narrowed after 2 h, and only few PCE polymers produced after 3 h; a similar trend was exhibited in Figure 3, Figure 5, Figure 6 and Figure 7.

The amounts of produced PCE polymer and consumed IPEG macromonomer of 52IPEG6.0 were slightly higher than that of 52IPEG5.0 and their mass fraction curves almost coincided in Figure 3 and Figure 7. This demonstrated that the steric hindrance of 52IPEG macromonomer would not influence its conversion when *A*/*E* was higher than 5.0. *A*/*E* improved the molecular weight of PCE polymers with a higher value according to Figure 2 and Figure 3, so the main chain length of PCE polymers in 52IPEG6.0 would be longer than 52IPEG5.0. The conversions of macromonomers in 52IPEG3.0 in first 2 h and 52IPEG6.0 in first 1 h were quite close, and their added ratios of acrylic acid were the same. It suggested that there were still sufficient macromonomers in 52IPEG6.0 in the first 2 h. The side chain density of 52IPEG3.0 and 52IPEG6.0 should be no significant difference in the period above, and their substantial difference was the main chain length which reflected in the molecular weight.

### 3.2. Molecular Properties Comparision

**Figure 8:**
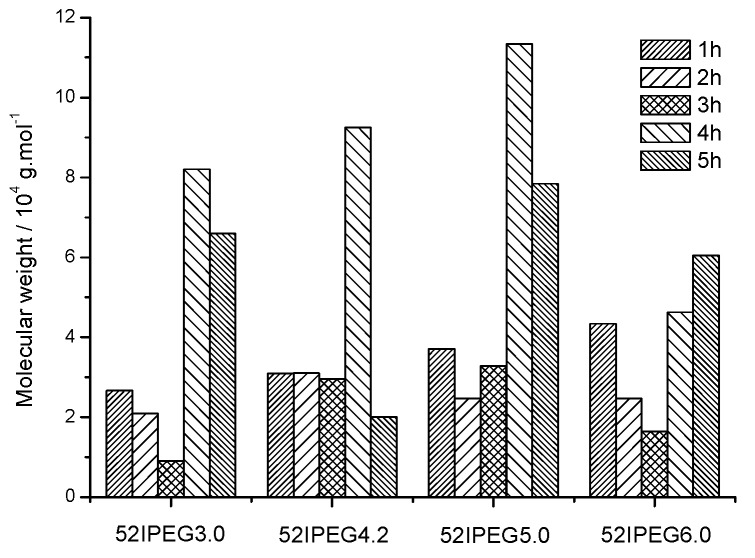
Molecular weight of 52IPEGs PCE polymers produced in different time intervals.

It was known to the skilled in this field that PCE samples contain polymers with different molecular weights. The general mass average molecular weight, mass fraction and number of components of a PCE sample were designated as $\bar{M_{w}}$, *W* and *i,* respectively. The mass average molecular weights of 52IPEGs polymers produced each hour could be calculated based on Formula (3) (Figure 2 and Figure 3). The results were presented in Figure 8.

(3) $$\bar{M_{w}}=\sum_{i}W_{i}M_{i}$$

Generally, the molecular weight of polymers with a time interval of 1 h decreased in the first 3 h, which agreed with the trend in Figure 2. PCE polymers with the highest *A*/*E* 6.0 and the lowest *A*/*E* 3.0 showed greater fluctuation for the molecular weights in different time intervals. The high *A*/*E* value increased the side density of polymers as well as its molecular weights during the adding of AA. Whereas there were few macromonomers left in the reaction system (except 52IPEG3.0) after 3 h, the target products should already have gained in the first 3 h. It would be energy conservation modification in the actual production once it was confirmed in application.

The ideal way to get a relatively uniform side chain density and main chain length PCE sample was to try to keep the constant *A*/*E* of dissociative monomers during the copolymerization. In this case, more acrylic acid should be added every half or 1 h on the decrease. The general molecular weights of polymers in 52IPEG4.2 in different time intervals were quite similar but possessed a decreasing side chain density. Take 52IPEG4.2, for example; its side chain density would increase if the adding amount of acrylic acid reduced in the 2nd h. The optimal polymers can be obtained after a series of trial and error.

The mass fraction of polymers with different molecular weights was presented in Table 2, which was derived by Figure 2 and Figure 3 and Formula (3). It reflects the exact polymerization procedures of PCE polymers.

According to the analysis of the molecular weight properties of Section 3.1 and Formulas (1) and (2), the structures of PCE polymers could be summarized as: (1) 52IPEG3.0 possessed the highest side chain density especially in the 1st hour; a certain amount polymers were still produced after 3 h; it possessed the shortest main chain length among 52IEPG series PCE samples. (2) 52IPEG4.2 exhibited increasing main chain length, decreasing side chain density and relative stable molecular weight during the reaction process; (3) 52IPEG5.0 possessed low side chain density and long main chain length; (4) 52IPEG6.0 exhibited the highest molecular weight, lowest side chain density and longest main chain length. The general *A/E* of IPEG3.0 and its molecular weight were much lower than other samples in the entire process. The dispersing effectiveness of 52IPEG3.0 polymers with particular structures could be deduced with the conclusion above and flow spread of cement paste [26,27]. The comparison of molecular structures of 52IPEGs PCE was shown in Table 3.

### 3.3. Dispersing Effectiveness of 52IPEGs

**Figure 9:**
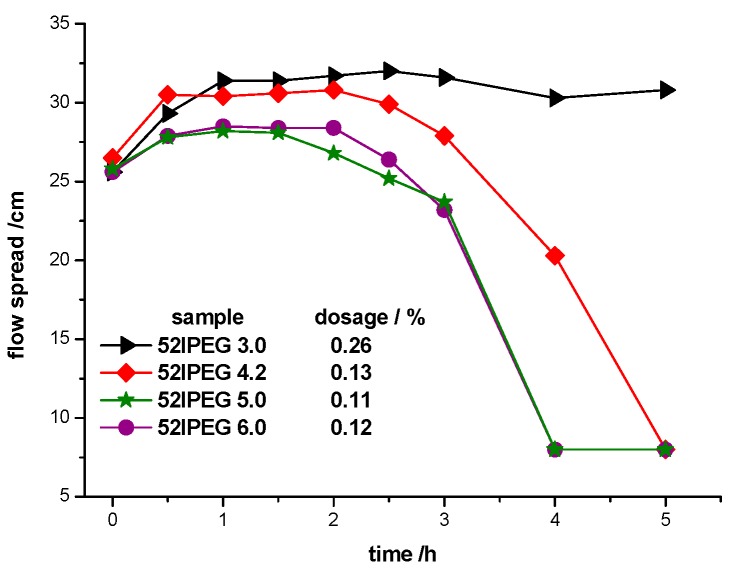
Mini slump results of cement paste added 52IPEG series PCEs.

The cement pasted added 52IPEG3.0 at an extremely high dosage of 0.26% by the weight of cement (bwc) kept high fluidity more than 5 h with the required initial 26 ± 0.5 cm flow spread according to Figure 9. The high side chain density provided strong steric hindrance on the surface of cement particles. Meanwhile, the low charge density of PCE polymers delayed their adsorption rate onto the cement particles [28,29]; thus, the polymers took longer time to achieve the adsorption balance.

52IPEG4.2, 52IPEG5.0 and 52IPEG6.0 achieved the flow spread of 26 ± 0.5 cm at the dosage around 0.12% bwc. There were two types of polymers with different structures contained in the 3 PCE samples. One type presented high molecular weight, high side chain density and short main chain length, which was produced earlier, providing the slump retention of cement paste; another type presented low molecular weight, low side chain density and long main chain length, providing high initial flow spread. It was obvious that PCE polymers with low side chain density provide initial dispersing effectiveness, and polymers with high side chain density offer the later retention ability.

## 4. Conclusions

The structures of PCE polymers synthesized at the *A*/*E* value of 3.0, 4.2, 5.0 and 6.0 were studied. The molecular weight, main chain length and reaction rate of PCE polymers as well as the conversion of macromonomers increased with the general adding ratio of acrylic acid to 52IPEG macromonomers. Polymers with a mass fraction of 53.8% were detected in 52IPEG6.0 after 1 h, and it was even higher than that of polymers of 52IPEG3.0 produced in the first 2 h.

The side chain density of polymers decreased with the general *A*/*E* value during the reaction process in a certain PCE sample. The continuous feeding of AA during the reaction was the reason that lowered the side chain density of polymers. PCE polymers produced in the 1st hour possessed the highest side chain density and molecular weight. It was the key period for the structure control of PCE polymers especially for high *A*/*E* value ones. The major effective polymers were produced during the adding of acrylic acid in the first 3 h.

High dosage of 0.26% for 52IPEG3.0 was needed to reach the required initial cement paste flow spread of 26 ± 0.5 cm, yet it exhibited excellent retention ability. Around only 0.12% dosages were needed for other PCE polymers, but their retention properties were not that good. 52IPEG4.2 possessed a molecular weight of around 30,000 g/mol and moderate side chain density; main chain length was a proper choice for the application. The structure of PCE polymers with certain properties could be designed based on influence factors.

## Figures and Tables

**Table 1:** Chemical and mineral compositions of CEM I 52.5 cement, weight fraction, %.

Composition	Results (%)
C_3_S, m	54.14
C_2_S, m	26.63
C_3_A, c	3.28
C_3_A, o	4.26
C_4_AF, o	2.45
Free lime	0.1
Periclase (MgO)	0.03
Anhydrite	2.64
CaSO_4_ hemihydrate	1.21
Gypsum	0.02
Calcite	3.61
Quartz	1.16
Arcanite (K_2_SO_4_)	0.46

**Table 2:** Mass fraction of 52IPEGs with different molecular weights produced each hour.

Sample	Molecular Weight (g/mol)					Mass Fraction (%)				
	1 h	2 h	3 h	4 h	5 h	1 h	2 h	3 h	4 h	5 h
52IPEG3.0	26,700	20,984	9007	82,043	65,945	28.5	22.1	9.0	9.0	3.8
52IPEG4.2	30,900	31,091	29,481	92,490	20,027	35.0	38.3	11.1	1.3	2.3
52IPEG5.0	37,103	24,681	32,900	413,429	78,419	52.2	26.7	9.8	0.14	1.6
52IPEG6.0	43,400	24,742	16,457	46,238	60,435	53.8	26.8	8.1	2.5	0.74

**Table 3:** Comparison of molecular structures of 52IPEGs.

Molecular Properties	52IPEG3.0	52IPEG4.2	52IPEG5.0	52IPEG6.0
Molecular weight	low	medium	high	high
Side chain density	high	medium	low	low
Main chain length	short	medium	long	long
